# Infection with the gastrointestinal nematode *Ostertagia ostertagi *in cattle affects mucus biosynthesis in the abomasum

**DOI:** 10.1186/1297-9716-42-61

**Published:** 2011-05-11

**Authors:** Manuela Rinaldi, Leentje Dreesen, Prisca R Hoorens, Robert W Li, Edwin Claerebout, Bruno Goddeeris, Jozef Vercruysse, Wim Van Den Broek, Peter Geldhof

**Affiliations:** 1 Department of Virology, Parasitology and Immunology, Faculty of Veterinary Medicine, Ghent University, Salisburylaan 133, 9820 Merelbeke, Belgium; 2 Bovine Functional Genomics Laboratory, Animal and Natural Resources Institute, USDA-ARS, 10300 Baltimore Avenue, Beltsville, MD 20705, USA; 3 Department of Biosystems, Faculty of Bioscience Engineering, K.U. Leuven, Kasteelpark Arenberg 30, 3001 Leuven, Belgium; 4 Department of Morphology, Faculty of Veterinary Medicine, Ghent University, Salisburylaan 133, 9820 Merelbeke, Belgium

## Abstract

The mucus layer in the gastrointestinal (GI) tract is considered to be the first line of defense to the external environment. Alteration in mucus components has been reported to occur during intestinal nematode infection in ruminants, but the role of mucus in response to abomasal parasites remains largely unclear. The aim of the current study was to analyze the effects of an *Ostertagia ostertagi *infection on the abomasal mucus biosynthesis in cattle. Increased gene expression of *MUC1*, *MUC6 *and *MUC20 *was observed, while *MUC5AC *did not change during infection. Qualitative changes of mucins, related to sugar composition, were also observed. AB-PAS and HID-AB stainings highlighted a decrease in neutral and an increase in acidic mucins, throughout the infection. Several genes involved in mucin core structure synthesis, branching and oligomerization, such as *GCNT3*, *GCNT4*, *A4GNT *and protein disulphide isomerases were found to be upregulated. Increase in mucin fucosylation was observed using the lectin UEA-I and through the evaluation of fucosyltransferases gene expression levels. Finally, transcription levels of 2 trefoil factors, *TFF1 *and *TFF3*, which are co-expressed with mucins in the GI tract, were also found to be significantly upregulated in infected animals. Although the alterations in mucus biosynthesis started early during infection, the biggest effects were found when adult worms were present on the surface of the abomasal mucosa and are likely caused by the alterations in mucosal cell populations, characterized by hyperplasia of mucus secreting cells.

## Introduction

The mucus layer in the gastrointestinal (GI) tract forms the first line of defense to the external environment. Mucins (MUC) or mucus glycoproteins are one of the most important components of the mucus barrier and they are classified as membrane bound and secreted mucins depending on their function and location. In cattle, 9 membrane-associated mucins and 8 secreted mucins have been identified [1]. Membrane bound mucins are mostly present on the apical membrane of epithelial cells, where they have been suggested to play a role in cell signaling, while secreted gel forming ones are capable of forming oligomers, crucial in the formation of the visco-elastic mucus gel [2]. Mucins are highly glycosylated proteins, with most of the oligosaccharides attached by O-links to the repetitive sequences rich in threonine and serine. Previous studies have suggested that mucin glycosylation not only protects the mucins from proteolytic enzymes, but it can also be altered in response to mucosal infection and inflammation, probably with protective effects [3]. The carbohydrate structures present on mucins are determined by the expression of specific glycosyltransferases. Thus mucin glycosylation is governed by tissue-specific enzyme expression, host and environmental factors influencing transferase expression [4].

Trefoil factors (TFF1, TFF2 and TFF3) are also major secretory products of normal mucus-secreting cells in the epithelium in the GI tract. In humans these factors are known to be co-expressed with mucins in mucus producing cells in the stomach and duodenum [5,6] and they seem to play a major role in wounding responses to maintain mucosal surface integrity, as well as in pathological processes [7,8]. It has been reported that TFF2, after addition to a mucin solution, significantly increases viscosity and elasticity of the mucus [8].

Although the precise role of mucus in host defense against nematode infections is not known, previous studies have shown that alteration in mucin production and glycosylation may be related to the capacity of the host to expel GI nematodes. For instance, increased Muc2 production in the intestine during infection with *Trichuris muris *was observed only in resistant mice and it was correlated with worm expulsion [9]. Moreover levels of Muc4, Muc13 and Muc17 were increased during acute and chronic infections with *T. muris*, causing thickening of the glycocalix in the intestine [10]. On the other hand, *MUC5AC *was found to be strongly downregulated in the abomasum of sheep infected with *Haemonchus contortus *[11], as well as in experimentally infected sheep selected for resistance to nematode infections [12]. In cattle, an infection with the intestinal nematode *Cooperia oncophora *also altered the mucus composition, causing induction of *MUC2 *and a downregulation of *MUC5B *in the small intestine [13]. Changes in terminal sugars of goblet cells mucins, such as a strong expression of terminal N-acetyl-D-galactosamine, were also observed in rats infected with the intestinal parasite *Nippostrongylus brasiliensis *around the time of immune-mediated expulsion [14]. Previous studies have also observed important changes in TFF expression during GI nematode infections. Rowe et al., [11] have reported an induction of TFF3 accompanied by a progressive loss of TFF2 in the abomasal mucosa of sheep infected with *H. contortus*. *TFF3 *was also found to be significantly upregulated in the small intestine of sheep infected with *Trichostrongylus columbriformis *[15] and in rats during infection with *N. brasiliensis *[16].

The abomasal nematode *Ostertagia ostertagi *causes a loss in animal production due to depression in food intake and impaired gastrointestinal functions [17]. The presence of larvae and adult worms in the abomasum is accompanied by morphological changes in the host mucosa, such as mucous cell hyperplasia, superficial epithelial damage and loss of acid-producing parietal cells [18-20]. To date little is known about the effects of these cellular changes on the composition of the abomasal mucus layer. The purpose of the current study was to analyze in more detail the effects of an *Ostertagia *infection in the abomasum on the mucus biosynthesis, i.e. mucins, TFFs, and a set of selected glycogenes and disulphide isomerases involved in the synthesis and oligomerization of mucins.

## Materials and methods

### Experimental design and tissue collection

Nematode-free Holstein calves, aged 6 to 8 months, were randomly selected and divided in experimental groups. The animals were kept indoors to prevent accidental infection with nematode parasites. The calves were fed hay and commercial pellets, and given *ad libitum *access to water. For gene transcription analyses, a trial including 4 groups of animals (*n *= 4 in each group) was performed (Trial 1). Animals sacrified at day 0 were used as negative controls, the remaining animals were orally infected with 100 000 *O. ostertagi *L3 larvae/animal and killed at 6, 9, and 24 days post infection (dpi). These time points corresponded with the presence of late L3/early L4, L4 and adult worm stages in the abomasum, respectively, as observed at slaughtering. For histological analyses, a second trial including three groups of animals (*n *= 3 in each group) was performed (Trial 2). Animals sacrified at day 0 were used as negative controls, the remaining animals were orally infected with 100 000 *O. ostertagi *L3 larvae/animal and killed at 14 and 21 dpi. One additional group of four animals was maintained for 60 days on pasture to acquire a natural *O. ostertagi *infection before euthanasia was performed at 60 days post exposure (dpe). Samples from these animals were collected for both gene transcription and histological analyses. For gene transcription analyses, tissue samples were collected from the fundic region of the abomasum, snap frozen in liquid nitrogen and stored at -80°C until RNA was extracted. For histological analyses abomasal tissues were stored in Carnoy's solution (60% ethanol, 30% chloroform, 10% glacial acetic acid) and in 10% formaldehyde in phosphate buffered saline (PBS), both freshly made. All experiments had been approved by the ethical committee of the Faculty of Veterinary Medicine at Ghent University.

### RNA extraction and cDNA synthesis

Total RNA was extracted from tissue samples using Trizol (Invitrogen) and further purified using the RNeasy Mini kit (Qiagen). To remove contaminating genomic DNA (gDNA), on-column DNase digestion was performed using the RNase-free DNase set (Qiagen) according to the manufacturer's instructions. RNA quality was verified using an Experion™Automated Electophoresis System (Bio-Rad), and concentrations were determined using a NanoDrop ND-1000 spectrophotometer (NanoDrop Technologies). Genomic DNA contamination was checked by the SuperScript One-Step RT PCR (Invitrogen) using intron-spanning primers for *gapdh *(Additional file 1).

### Quantitative Real-time PCR

Quantitative Real-time PCR (qRT-PCR) was performed for the following genes (Additional file 1 Table S1): mucins (*MUC1*, *MUC2*, *MUC5AC*, *MUC5B*, *MUC6*, *MUC20*); trefoil factors (*TFF1*, *TFF2*, *TFF3*); glycosyltransferases [alpha-1, 4-N-acetylglucosaminyltransferase (*A4GNT*); betaGal beta-1,3-N-acetylglucosaminyltransferase 3 (*B3GNT3*); core 1 synthase, glycoprotein-N-acetylgalactosamine 3-beta-galactosyltransferase 1 (*C1GALT1*); glucosaminyl (N-acetyl) transferase 2, I-branching enzyme (I blood group) (*GCNT2*); glucosaminyl (N-acetyl) transferase 3, mucin type (*GCNT3*); glucosaminnyl (N-acetyl) transferase 4, core 2 (*GCNT4*)]; sulfotransferases [heparan sulfate (glucosamine) 3-O-sulfotransferase 1 (*HS3ST1*); heparan sulfate (glucosamine) 3-O-sulfotransferase 2 (*HS3ST2*); galactose-3-O-sulfotransferase 1 (*GAL3ST1*); sialyltransferases [ST3 beta-galactoside alpha-2,3-sialyltransferase 4 (*ST3GAL4*)]; fucosyltransferases (*FUT1*, *FUT2*, *FUT4*, *FUT10*); and disulphide isomerases [anterior gradient homolog 2 (*AGR2*); protein disulfide isomerase family A, member 3 (*PDIA3*); protein disulfide isomerase family A, member 4 (*PDIA4*)].

One μg of total RNA was converted to cDNA using the iScript cDNA synthesis kit (Bio-Rad), following the manufacturer's instructions. Real-time-PCR analyses was carried out with the SYBR Green Master Mix (Applied Biosystems) using 400 nM of each amplification primer and 2 μL of single-stranded cDNA (10 ng of the input total RNA equivalent) in a 20 μL reaction volume. Amplification cycles were performed on a StepOnePlus Real-Time PCR System (Applied Biosystems) under the following conditions: 95°C for 20 s followed by 35 cycles of 95°C for 5 s and optimal annealing temperature (Ta) for 30 s (Additional file 1). The primer sets used to amplify the different genes were designed using the Primer3 software http://frodo.wi.mit.edu/primer3/ and are listed in Additional file 1. Reaction efficiencies were measured based on a standard curve using dilution series of pooled cDNA from all the samples. A melting curve analysis was performed at the end of the reaction to ensure specificity of the primers. In addition, PCR products were cloned in the *pGEM-T vector *according to the manufacturer's instruction (Promega) and sequenced. Every assay included cDNA samples in duplicate and a non-template control. Ct values were transformed in relative quantity using the delta Ct method applying the formula: Q = E ^(min Ct - sample Ct)^, with Q = sample quantity relative to sample with highest expression, E = amplification efficiency, and min Ct = lowest Ct value = Ct value of sample with the highest expression level. To minimize technical mistakes and therefore misinterpretation of results, qRT-PCRs for each gene were carried out on the same plate with all samples.

Statistical analysis was carried out using GraphPad Prism software. The Nonparametric Mann Whitney U test was used to determine differences between infected and control groups. A *P*-value of ≤ 0,05 was considered significant.

### Histochemistry

Immediately after collection, tissue samples were placed in Carnoy's solution for 24 h at room temperature (RT) and then in 70% ethanol at RT. The samples were then dehydrated through a graded ethanol, cleared in xylene, and embedded in paraffin. For histochemical examination, serial paraffin sections were cut at 8 μm thickness. Tissue sections were deparaffinized in xylene and isopropanol followed by rehydration through a graded ethanol series and stained with periodic acid Schiff (PAS) [21], Alcian blue (AB)-PAS (pH 2.5) [22,23] and High iron diamine (HID)-AB stainings as previously described [24] with slight modifications. Briefly, for PAS staining samples were incubated with periodic acid for 10 min, washed, and covered with Schiff's solution (Merck) for 20 min. A wash in sulfite water (12 mL Na_2_S_2_O_5 _+ 10 mL HCl + 200 mL distilled water) for 15 min was followed by counterstain with hematoxylin. For the AB-PAS staining, samples were incubated in AB solution for 15 min, rinsed in water and incubated with Schiff's solution for 15 min. Following a 15 min wash in sulfite water they were counterstained with hematoxylin. Finally for the HID-AB staining, sections were incubated in diamine solution for 16 h and then counterstained with 1% AB in 3% acetic acid for 10 min. All the sections were then dehydrated in ethanol, cleared in xylene and mounted in synthetic medium for observation using light microscopy. Sections from control (0 dpi; *n *= 3) and infected animals (14 and 21 dpi, 60 dpe; *n *= 3) were used. Three pictures for each section were taken and analyzed.

### Immunohistochemistry for TFF3

Immediately after collection, tissue samples were placed in 10% formaldehyde for 24 h at RT followed by distilled water for 1 h at RT and then 70% ethanol at RT. The samples were then dehydrated through a graded ethanol, cleared in xylene, and embedded in paraffin. Serial paraffin sections were cut at 5 μm thickness and mounted onto APES-coated glass slides. Tissue sections were deparaffinized in xylene and isopropanol, followed by rehydration through a graded ethanol series and immunostained with anti-hTFF3 (rabbit polyclonal, 1:100, Santa Cruz Biotechnology). Antigen retrieval was performed by incubating the slides in citrate buffer, microwave heating, and cooling for 30 min at 4°C. Non specific staining was blocked with 1% of BSA and 0.3% Triton X-100 pH 7.5 (Sigma) in PBS for 15 min at RT, followed by incubation with 1% of BSA in PBS for 30 min at RT. Sections were subsequentely incubated overnight at 4°C with the primary antibody diluted in 1% BSA in PBS. Slides were covered with Alexa Fluor^® ^488 goat anti-rabbit IgG (H+L) (Invitrogen) for 1 h at RT. Counterstaining was performed with DAPI (4',6-diamidino-2-phenylindole, dilactate; 1:1000 in PBS; Invitrogen) for 5 min at RT. Then sections were dehydrated in ethanol, cleared in xylene and mounted in synthetic medium for observation using fluorescent microscopy. Negative control stainings were carried out using the same procedure except that the primary antibody was omitted and replaced with buffer. Sections from control (0 dpi; *n *= 4) and infected animals (60 dpe; *n *= 4) were used. Three pictures for each section were taken and analyzed.

### Lectin histochemistry

Samples used for lectin histochemistry were fixed in Carnoy's solution and serial paraffin sections were cut at 5 μm thickness. Sections were then deparaffinized in xylene and isopropanol followed by rehydration through a graded ethanol series. They were stained with a panel of six lectins (details in Table 1), following the manifacturer's instruction using a lectin-biotin avidin-peroxidase method (Vectastain elite ABC kit), with DAB (Vectastain) as the disclosing agent and a haematoxylin counterstain. Briefly the endogenous peroxidase activity was blocked with 3% H_2_O_2 _in distilled water for 5 min. Non specific staining was blocked with 1% of BSA in PBS for 10 min at RT. Biotin labeled lectins (Vectastain) were diluted in lectin buffer (1 mM Hepes, 150 mM NaCl, 1 mM CaCl, 1 mM MgCl in PBS) to the desired concentration (Table 1) and incubated for 1 h at RT. Negative control sections were included in every staining run with buffer replacing the lectin. Specificity of lectin binding was checked by pre-incubating each lectin with the appropriate concentration of the competing sugar (Table 1) for 1 h at RT. Sections from control (0 dpi; *n *= 3) and infected animals (14 and 21 dpi, 60 dpe; *n *= 3) were used. Three pictures for each section were taken and staining intensity was ranked using (-) for absent, (+/-) for just detectable, (+) for positive and (++) for strongly positive staining as reported in Table 2.

**Table 1:** The origins and sugar binding properties of the lectins used in this study.

Lectin origine	Acronym (μg/ml)^a^	Sugar specificity	Inhibitor ^b ^(mM)^c^
*Arachis hypogaea*	PNA (1)	Galβ1,3GalNAcα1-> Galβ1,4GlcNAcβ1-	Gal (200)
*Dolichos biflorus*	DBA (1)	GalNAcα1,3(LFucα1,2)Galβ1,3/4GlcNAcβ1-	GalNAc (200)
*Glycine max*	SBA (1)	GalNAcα1,3->Galα1	GalNAc (200)
*Triticum vulgaris*	WGA (0.1)	GlcNAcβ1,4	HOAc (100)
*Ulex europaeus*	UEA-I (1)	α-L-Fucosyl terminals	I-Fuc (200)
*Ricinus comunis*	RCA _120 _(1)	Galβ1,4GlcNAc->β-Gal-	Gal (200)

^a^(μg/mL): concentration of the lectins used in the study.

^b^Inhibitor: Gal (galactose), GalNAc (N-Acetylgalactosamine), HOAc (acetic acid), I-Fuc (I-fucose).

^c^(mM): concentration of the competing sugar used in the study.

**Table 2:** Lectin staining, subjectively described from ++ (very strong) to - (absent) during a primary infection with *O.ostertagi*.

lectin	time (dpi/dpe)^a^	mucus^b^	SMC^c^	MNC^d^
**UEAI**	0	+/-	-	++
	14	+/-	+/-	++
	21	+	+	++
	60	+	+	++
				
**WGA**	0	++	++	++
	14	++	++	++
	21	+	+	+
	60	+	+	+
				
**RCA120**	0	++	++	++
	14	++	++	++
	21	++	++	+
	60	++	++	++
				
**DBA**	0	++	++	++
	14	++	++	++
	21	++	++	++
	60	+	+	++
				
**SBA**	0	++	++	++
	14	++	+	++
	21	++	++	++
	60	++	++	++
				
**PNA**	0	+	++	++
	14	+	+	++
	21	+/-	+/-	+/-
	60	+	+	++

UEA-I binds L-fucose, WGA binds N-acetyl glucosamine, RCA_120_, DBA, SBA and PNA belong to the galactose/N-acetylgalactosamine binding lectins.

^a^dpi: days post infection (14, 21) dpe: days post exposure (60).

^b^mucus: extracellular mucus on the surface of the epithelial cells.

^c^SMCs: surface mucus cells.

^d^MNCs: mucus neck cells.

## Results

### Alteration of mucus biosynthesis during *Ostertagia *infection

The results of the quantitative Real-Time PCR (qRT-PCR) analyses on components and regulatory enzymes of the mucus layer during an infection with *O. ostertagi *are shown in Table 3. Among the membrane bound and secreted mucins tested, *MUC1*, *MUC20 *and *MUC6 *transcription levels were observed to increase during the infection. The peak of induction was reached at 24 dpi. Similar results were observed in animals maintained on pasture for 60 days. No changes were observed in *MUC5AC *transcription level during infection. Significant increases were also observed for *TFF1 *and *TFF3*, with an increase of ~ 60 fold for *TFF3 *at 24 dpi. Both *TFF1 *and *TFF3 *were also upregulated in animals at 60 days post-exposure (dpe). For the glycosyltransferases involved in mucin biosynthesis, *GCNT3 *and *GCNT4*, both involved in the synthesis of core structures, were found to be up-regulated during infection, while *C1GALT1 *a key enzyme for the core 1 formation was not affected by the infection. *GCNT2 *and *A4GNT*, both branching O-glycans enzymes, were already upregulated 6 dpi. *GCNT2 *peaked, with ~12 fold change compared to control, at 24 dpi. It was also significantly upregulated, as well as *A4GNT*, at 60 dpe. These animals also had increased transcription levels of the enzyme *B3GNT3 *compared to uninfected animals. Several sulfotransferases and a sialyltransferase were also tested, but only *HS3ST1 *and *ST3GAL4 *were detected in the abomasum and their transcription level did not change during infection. The fucosyltransferases *FUT2 *and *FUT4 *were both up-regulated at 24 dpi and 60 dpe with an increase of ~3 fold at both time points. Finally, three disulphide isomerases were tested i.e. *AGR2*, *PDIA3 *and *PDIA4*. They were all found to be impacted throughout the infection starting from 6 dpi. The up-regulation peaked at 24 dpi with fold changes varying between 4.12 and 6.95. Increased transcription levels were also observed for these three enzymes at 60 dpe.

**Table 3:** Transcription profile of genes during a primary infection with *O.ostertagi*

Gene			Infection^b^	
	**6 dpi**	**9 dpi**	**24 dpi**	**60 dpe**
	
**Mucins**				
**Membrane bound**				
MUC1	2.07 ± 0.3*	2.93 ± 0.3*	3.29 ± 0.3*	2.75 ± 0.2*
MUC20	2.78 ± 0.5*	1.50 ± 0.2*	3.99 ± 0.6*	3.28 ± 0.6*
**Secreted gel forming**				
MUC2	n.d^c^	n.d	n.d	n.d
MUC5AC	4.08 ± 1.1*	2.25 ± 0.5*	1.50 ± 0.2*	1.56 ± 0.7*
MUC5B	n.d	n.d	n.d	n.d
MUC6	3.09 ± 1.0*	2.80 ± 0.4*	4.39 ± 1.4*	4.52 ± 0.5*
				
**Trefoil Factors**				
TFF1	3.89 ± 1.5*	3.32 ± 0.8*	2.57 ± 0.2*	3.90 ± 0.5*
TFF2	2.50 ± 1.1*	4.12 ± 2.4*	2.22 ± 0.8*	3.81 ± 1.4*
TFF3	2.59 ± 0.8*	18.57 ± 11.7*	58.57 ± 13.2*	49.28 ± 16.7*
				
**Glycosyltransferases**				
**Core structure**				
GCNT3	2.93 ± 0.6*	3.73 ± 0.3*	5.90 ± 1.0*	4.29 ± 0.6*
GCNT4	2.92 ± 0.3*	2.33 ± 0.2*	3.02 ± 0.2*	3.14 ± 0.3*
C1GALT1	3.06 ± 0.6*	2.09 ± 0.2*	2.22 ± 0.5*	2.35 ± 0.2*
**Backbone and terminal**				
GCNT2	2.87 ± 0.8*	1.60 ± 0.2	12.44 ± 4.2*	6.74 ± 1.0*
A4GNT	3.32 ± 0.3*	2.03 ± 0.3	3.12 ± 0.8*	2.75 ± 0.1*
B3GNT3	4.51 ± 1.1*	3.11 ± 0.6*	2.57 ± 0.8*	4.61 ± 0.4*
				
**Sulfotransferases**				
H3ST1	1.14 ± 0.2*	2.10 ± 0.6*	4.52 ± 2.6*	2.35 ± 0.5*
H3ST2	n.d	n.d	n.d	n.d
GAL3ST1	n.d	n.d	n.d	n.d
				
**Sialyltransferases**				
ST3GAL4	1.60 ± 0.1*	1.20 ± 0.2*	1.89 ± 0.3*	1.15 ± 0.1*
				
**Fucosyltransferases**				
FUT1	1.94 ± 0.5*	1.27 ± 0.2*	0.77 ± 0.1*	1.33 ± 0.1*
FUT2	2.35 ± .4*	2.56 ± 0.8*	3.13 ± 0.4*	3.15 ± 0.2*
FUT4	2.77 ± 0.5*	2.89 ± 1.0*	3.09 ± 0.5*	3.13 ± 0.6*
FUT10	1.65 ± 0.2*	1.47 ± 0.2*	1.46 ± 0.2*	1.58 ± 0.1*
				
**Disulphide isomerase**				
AGR2	3.41 ± 0.6*	3.44 ± 0.4*	5.82 ± 0.9*	5.24 ± 0.8*
PDIA3	3.16 ± 0.3*	2.60 ± 0.4*	4.12 ± 1.0*	4.46 ± 0.6*
PDIA4	2.58 ± 0.2*	3.51 ± 0.8*	6.95 ± 0.9*	5.37 ± 0.9*

^a^

^a^mean ± error standard of fold changes in infected animals compared to control (0 dpi).

^b^dpi: days post-infection dpe: days post-exposure.

^c^n.d.: not determined, too low in the sample.

*p value ≤ 0.05 control 0 dpi vs infected animals (6, 9, 24 dpi; 60 dpe).

### Alteration of neutral and acidic mucins during *Ostertagia *infection

**Figure 1:**
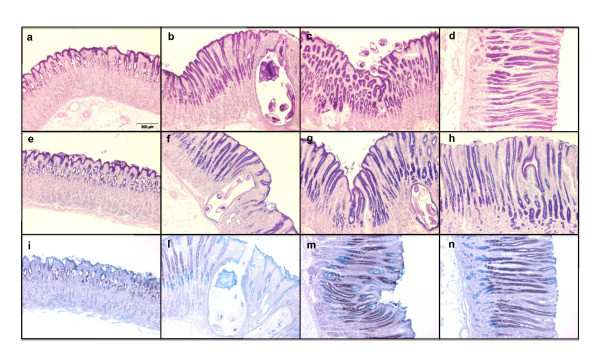
**Histochemical staining of Carnoy's fixed, paraffin-embedded abomasum sections of Holstein cows after Ostertagia ostertagi infection**. PAS (A-D) stained mucins in pink; AB-PAS pH 2.5 (E-H) stained neutral mucins in magenta and acidic mucins in blue; HID-AB (I-N) stained sialylated mucins in blue and sulphated mucins in brown. (A, E, I) negative control, (B, F, L) infected for 14 days, (C, G, M) infected for 21 days, (D, H, N) exposed for 60 days. Original magnification 10×.

Histochemistry was performed to detect mucins in the mucosa of the abomasum during an infection with *O. ostertagi *(Figure 1). PAS, which stains all glycoproteins including mucins, was observed in surface mucus cells (SMCs) and mucus neck cells (MNCs) of uninfected animals (Figure 1a). At 14 dpi a lower presence of mucins was observed in the infected glands (Figure 1b). At 21 dpi and at 60 dpe mucins re-emerged in the SMCs and MNCs to similar levels as in control animals. AB-PAS pH 2.5 stains neutral mucins in magenta, acidic mucins in blue and a mixture of the two in purple. In control animals (Figure 1e), the SMCs were found to produce a mixture of neutral and acidic mucins on the top of the fundic folds and in the gastric pits. Deeper in the gland, MNCs produced only acidic mucins. During infection, at 14 dpi, loss of staining was observed around the infected glands, while in the rest of the mucosa both the SMCs and MNCs produced a mixture of neutral and acidic mucins (Figure 1f). Similar results were obtained for the samples collected 21 dpi and 60 dpe (Figure 1g-h). Finally HID-AB staining for acidic mucins was performed, coloring the sialylated in blue and the sulfated in brown. In uninfected animals, sialylated mucins were identified in the superficial layer of the mucosa (SMCs) and a mixture of sulfated and sialylated in the gastric pits, while only sulfated mucins were observed in the neck region (MNCs) (Figure 1i). At 14 dpi (Figure 1l), less staining around the infected gland was observed. At 14 (Figure 1l), 21 dpi (Figure 1m) and at 60 dpe (Figure 1n) MNCs appear to produce not only sulfated mucins, as in control animals, but also sialylated ones.

### Immunohistochemistry for TFF3

**Figure 2:**
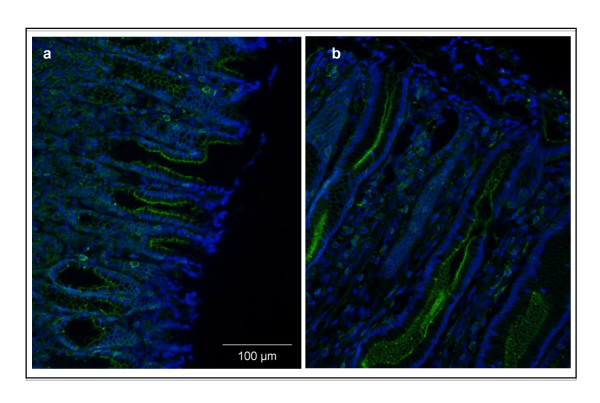
**Trefoil factor 3 immunofluorescent staining (green), combined with DAPI staining (blue)**. Formalin fixed, paraffin-embedded abomasum sections of Holstein cows after *Ostertagia ostertagi *infection were stained. (A) negative control, (B) exposed to *O. ostertagi *for 60 days. Original magnification 20×.

In uninfected animals (Figure 2a), TFF3 (green fluorescence) was expressed in the SMCs. A stronger staining was observed at 60 dpe (Figure 2b) in the hyperplastic gastric glands, produced by mucus epithelial cells.

### Alteration in the mucin saccharide residues during *Ostertagia *infection

**Figure 3:**
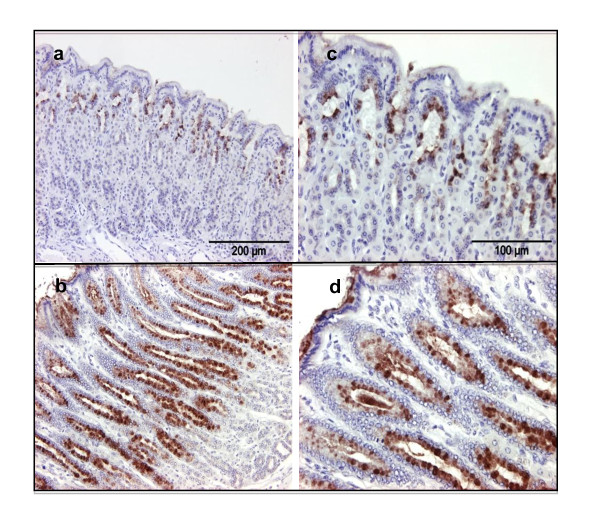
**Lectin staining with UEAI (a-L-Fucosyl terminals)**. Carnoy's fixed, paraffin-embedded abomasum sections of Holstein cows after *Ostertagia ostertagi *infection were stained. (A and C) negative control, (B and D) exposed to *O. ostertagi *for 21 days. Original magnification 20× (left panels), 40× (right panels).

To study alterations in the saccharide residues on mucins, 6 lectins were used to stain the abomasal mucosa of control (0 dpi) and infected (14, 21 dpi and 60 dpe) animals (Table 1). Both the secreted mucus and the mucus producing cells (SMCs and MNCs) of healthy animals were strongly (++) stained by WGA, which binds N-acetyl glucosamine and by RCA_120_, DBA, SBA and PNA, which all have high affinity for galactose and N-acetylgalactosamine residues (Table 2). UEA-I, a lectin with affinity for L-fucose, stained the MNCs (++) intensely in uninfected animals whereas SMCs were not stained (-) and the secreted mucus only stained weakly (+/-) (Table 2). The staining pattern of only two lectins was found to be modified after an *O. ostertagi *infection. UEA-I (for L-Fuc) showed an increased staining of secreted mucus and SMCs at 21 dpi (Figure 3b and 3d) and 60 dpe (Figure 3a and 3c). Conversely, PNA staining (for Gal and GalNAc) was decreased at 21 dpi in secreted mucus, SMCs and MNCs (Table 2). The other lectins did not seem to give an altered staining during infection (Table 2).

## Discussion

The importance of mucus as a host defensive mechanism against intestinal nematode infections has been reported in several animal species, including sheep, cattle and rodents [13,15,25,26], but the role of mucus in the response to abomasal parasites remains largely unclear. Apart from the recently reported changes in *TFF3*, *GCNT3 *and gastrokine 2 transcription levels during an *O. ostertagi *infection [27], little is known about the effect of this parasite on abomasal mucus composition and synthesis.

In the current study, increased gene expression of mucins, which are normally present in the abomasum, was observed for *MUC1*, *MUC6 *and *MUC20*. Although the transcriptional upregulation started early during infection, the highest changes were found when adult worms were present on the surface of the abomasal mucosa, after emergence from the infected glands. Transcription level of *MUC5AC*, another mucin normally present in abomasal tissues, did not change during infection. Previous studies in humans have shown that the gastric mucus layer is able to react to invading pathogens, such as *Helicobacter pylori*, through the modification of the expression profile of MUC1, MUC5AC and MUC6, but the role of these changes still remains unknown [28-30].

Qualitative changes of mucins, related to sugar composition, were also observed. Histochemistry of neutral and acidic (sialylated and sulfated) mucins in uninfected animals was found to be similar to what has been described in previous studies in abomasum of sheep [31]. Consistent with previous observations [19,32], the abomasal mucosa was found to be thickened at 24 dpi and in animals exposed for 60 days to a natural infection, compared to uninfected control animals. The length of the fundic glands was increased, due to the hyperplasia of the mucus secreting epithelium, as shown by PAS staining. The hyperplasia of these mucus-secreting cells may be the reason for the detected increase in mucin gene transcription levels during the infection. At 14 dpi, AB-PAS and HID-AB stainings showed that the abomasal mucosa was characterized by depletion of both neutral and acidic mucins, confined to the area invaded by the parasite larvae, indicating a strictly localized response to the parasite. The limited modification observed at this stage of the infection may be an attempt of the parasite to create an optimal environment for itself through the production of enzymes that break down mucin protein and carbohydrate structures, as previously suggested [33]. In the rest of the mucosa, during the infection, the alteration in mucin composition, in particular in the hyperplastic glands, was characterized by a decrease of neutral and an increase in acidic mucins produced by the MNCs in the neck region of the gland. These alterations in mucin composition are very similar to what has been described in sheep during a primary infection with *H. contortus *[31]. Since the modification of the glycosylation status of mucins has been reported to relate with alteration in the viscoelastic properties of mucus [34,35] and the possible attachment of the parasite [16,31], these alterations may be an attempt of the host to eliminate the parasite.

Genes involved in mucin core structure synthesis and branching were observed to be altered in infected animals compared to controls. Biosynthesis of mucin O-linked glycan is a complicated process, which starts with the formation of N-acetylgalactosamine (GalNAC), followed by the synthesis of four main core structures (core 1, 2, 3 and 4) that can be branched to form a high variety of glycans. GCNT enzymes are involved in these processes and among the genes analyzed, *GCNT3 *and *GCNT4 *were found to be upregulated. Interestingly, in a previous study on cattle infected with *C. oncophora*, strong upregulation of *GCNT3 *in intestinal goblet cells and in columnar epithelial cell was noticed throughout the infection. As observed in the current study, the increased transcription level started early during the infection (6 dpi). GCNT3 catalyzes a key rate-limiting step in mucins biosynthesis. An early upregulation during nematode infections suggests an enhancement in mucin secretion and an early capacity of the host to respond to the presence of the parasite, before the major alterations and damages in the mucosa appear, independently from the species of the invading parasite. During infection with *O. ostertagi *some of the sugar residues of the mucins in the abomasal mucosa were also found to be altered. Staining with the UEA-I lectin, which binds to α-L-Fucosyl residues, was increased during infection, in particular in the secreted mucus and in SMCs, compared to control animals. This observation is in contrast with the results of Hoang et al. [36] where a reduced UEA binding was observed in the abomasal fundus during infection with *T. circumcincta*. Almost all fucose in mucus is found on mucins where it has an important effect on the viscosity of the mucus [34,37]. The increased levels of fucose in the tissue during infection are consistent with the observed increase in transcription levels of *FUT2 *and *FUT4*, coding for enzymes transfering Fuc α-1,2 and Fuc α-1,3 respectively, on mucins. Similarly in the small intestine of rats infected with *N. brasiliensis*, an induced fucosylation, due to an upregulation of Fut2 gene expression, has been reported [38]. Disulphide isomerases are a family of enzymes that play an important role in the process of disulphide bond formation of gel forming mucins [39,40]. They are expressed in several tissues, including the stomach and the intestine where they are produced by mucus secreting cells [40,41]. Increased transcription levels of 3 disulphide isomerases (*AGR2*, *PDIA3 *and *PDIA4*) were observed after an *O. ostertagi *infection. Since the polymerization of mucin monomers is crucial in the formation of viscoelastic mucus, an increase of disulphide isomerases level may increase the gastric mucus viscosity. Although further studies analyzing the rheological properties of mucus during infection need to be done, it is possible that all the modification observed in mucus may be related to an attempt of the host to eliminate the invading pathogens.

Trefoil factor peptides are normally synthesized and secreted in human gastric and intestinal mucosa [42]. In humans, TFF1 is predominantly located in the foveolar cells of the gastric mucosa, TFF2 in the MNCs and deep in the pyloric glands, while TFF3, also called intestinal trefoil factor, is expressed mainly by goblet cells of the large and small intestine [43,44]. TFF3 has recently also been localized in the human gastric cardia [45]. In the current study, a strong upregulation of *TFF3 *was observed from 9 dpi onward. Immunofluorescence confirmed the increased expression of TFF3 in the mucosa of infected animals and showed that SMCs produce this peptide in the bovine fundus of uninfected calves, similar to what has been described in man [45]. In infected animals the hyperplastic mucus secreting cells appear to be the ones producing TFF3. The TFF3 upregulation is consistent with a recent study of Li et al. [27] that have showed an increase of *TFF3 *mRNA levels in primary and repeated infections with *O. ostertagi*. *TFF3 *upregulation has also been observed during *H. contortus *and *T. colubriformis *infections in sheep [15]. *TFF1 *was also observed to be upregulated at 24 dpi. To our knowledge this is the first time that *TFF1 *upregulation is observed during a GI nematode infection. In man, gastric pit cells (SMCs) in the cardia are able to synthesize TFF1, TFF3 and MUC5AC [45]. It is possible that the hyperplastic TFF3-expressing cells in the abomasum of infected calves also produce TFF1. The potential role of TFF3 during nematode infections has been related to mucosal defense and tissue restitution [15,27] but it still remains unknown if TFF1 may also contribute to tissue repair.

In conclusion, this study has shown that the abomasal mucin, TFFs and glycogenes transcription levels, as well as mucin glycosylation patterns are significantly altered during an *Ostertagia *infection in cattle. These changes are likely caused by the alterations in mucosal cell populations, characterized by hyperplasia of mucus secreting cells. The effect of these changes on the protective mucus barrier is still unclear. Studies on host immune response against *O. ostertagi *have highlighted that expulsion of adult worms during primary infection is uncommon [46,47], therefore alteration in mucin sugar composition and mucus viscosity does not seem to be an efficient method to eliminate this parasite from the abomasum in this stage of the infection. Further studies comparing the mucosal changes in primary infected animals versus animals with an acquired immunity against *O. ostertagi*, will be helpful in clarifying if mucus has a protective role against this parasite.

## Competing interests

The authors declare that they have no competing interests.

## Authors' contributions

MR had the main responsibility for the whole study, including lab work, analyzing and interpreting data, as well as writing the manuscript. PRH and RL performed the trials and collected the tissue samples. LD helped in collecting samples and performed the lectin staining and the immunohistochemistry staining. WV participated in the staining work. EC, BG, JV and PG conceived and designed the project and participated in the interpretation and discussions regarding the results, as well as in the writing of the manuscript. All authors read and approved the final manuscript.

## Supplementary Material

Additional file 1 **Table S1**. Primer sequences, amplicon length and annealing temperature of genes analyzed using quantitative Real Time PCR. Click here for file
